# Using an informed consent in mammography screening: a randomized trial

**DOI:** 10.1002/cam4.525

**Published:** 2015-09-17

**Authors:** José M. Baena‐Cañada, Petra Rosado‐Varela, Inmaculada Expósito‐Álvarez, Macarena González‐Guerrero, Juan Nieto‐Vera, Encarnación Benítez‐Rodríguez

**Affiliations:** ^1^ Medical Oncology ServicePuerta del Mar University HospitalCádizSpain; ^2^ Epidemiology, Prevention Unit, Health Surveillance and PromotionHealth District Bay of Cadiz – La JandaCádizSpain; ^3^ Population Cancer RegistryProvincial Office of HealthCádizSpain

**Keywords:** Breast imaging, breast tumors, diagnostic radiology, medical ethics, preventive medicine, screening mammography

## Abstract

Spanish women do not make an informed choice regarding breast cancer screening (BCS). Our aim was to evaluate the impact of receiving information regarding real BCS benefits and risks on knowledge, attitude, decision, feelings, and worries about cancer. Randomized controlled clinical trial of 355 women aged between 45 and 67 years, 177 and 178 assigned to the intervention group (IG) and control group (CG), respectively. After breast screening, women received either Nordic Cochrane Centre information on BCS or standard information. The primary outcome (knowledge) was determined from questionnaire administered at baseline and after a month. Answers were scored from 0 to 10 and scores of 5 or more indicated that women were well informed (had “good knowledge”). Questionnaires regarding attitudes, future screening intentions, and psychosocial impact were also administered. The Chi‐squared and Student's *t*‐tests were used to compare qualitative and quantitative variables, respectively. Good knowledge was acquired by 32 (18.10%) IG women and 15 (8.40%) CG women (*P* = 0.008). Mean scores from first to second interview increased from 2.97 (SD 1.16) to 3.43 (SD 1.39) in the CG and from and from 2.96 (SD 1.23) to 3.95 (SD 1.78) (*P *= 0.002) in the IG. No differences were found in the secondary endpoints. Women receiving information based on the Nordic Cochrane Centre document were better informed. This means of providing information is not very efficacious, nor does it modify attitude, decision, feelings, or worries about cancer.

## Introduction

The various sources available to Spanish women for accurate information on breast cancer screening (BCS) by mammography – healthcare professionals, communications media, informative documents, and websites, are not fully informative 1; they are aimed more at obtaining a high rate of participation than ensuring an informed choice [2, 3]. In a study on the content of the written information provided under each of the nineteen programs for BCS in Spain it was concluded that there is almost no information at all on the risks (i.e., false positive and false negative results, overdiagnosis and overtreatment, and radiation) 3 and in none of the programs is reference made to informed consent. The reality is that the amount and quality of knowledge that women demonstrate having is very deficient [4, 5]. More than 90% of women either do not know or else over‐estimate the benefit in reduction of mortality by participating in mammography 4. Very few are aware of the potential damage to them from overdiagnosis and overtreatment, nor do they perceive that false positives may harm them psychologically [5, 6].

The customary way of informing women about the screening program and proposing participation is by printed material sent with the personalized invitation letter. In previous randomized trials [7, 8] it has been demonstrated that an informative leaflet did help women learn more about breast cancer and screening, but, in general, the evaluation of informative leaflets in use has found them unsatisfactory, lacking the information essential for taking an informed decision. Accurate information on secondary effects and risks would require substantial improvement to these leaflets 9, 10, 11. Ideally there would be qualified personnel assigned to screening programs who would inform prospective users directly and in person. However, as screening programs are presently organized, this does not appear to be feasible; one proposal is to seek informed consent by sampling of the community 12.

The hypothesis formulated for our study is that providing accurate verbal and written information on the benefits and risks of mammography screening by a healthcare professional would increase the knowledge of prospective participants, and would modify their attitude and decision on whether or not to participate without increasing their anxiety, depression, and worries about cancer.

## Material and Methods

### Participants

Study participants were women resident in the Cadiz‐La Janda Health District, aged 45–67 years, who had been called to an examination by mammography under the BCS Programme, and had attended. They were required to have the capacity to give their informed consent to participate in the study; they should not have personal history of breast cancer and should not have participated in the last mammography examination as being 68–69 years old. Participants were recruited between January 2011 and September 2012. The study was registered (ClinicalTrials.gov Identifier: NCT01335906) in April 2011.

### Ethics statement

Prior to the study, research processes and materials were reviewed and approved by the Clinical Research Ethics Committee of the Hospital Universitario Puerta del Mar de Cádiz (PI‐0315‐2010).

### Interventions

After a mammography was performed, women were informed about the study and gave their informed written consent to participating. The women were not informed prior to the mammogram so as not to interfere in any way with their decision to participate. Interviews with a single researcher (either of the doctors or the nurse) took approximately 20 min. In this first interview personal identification data were obtained before the patients completed specific questionnaires to ascertain their knowledge, attitudes, decision, anxiety, depression, and fears of cancer.

Participants were next randomly assigned to either the group receiving standard information or the group receiving the experimental information. The women in both groups had received a letter, by normal post, which essentially gave them the instructions to follow and the date for their attendance to be examined (see Data S1). This letter comprised two pages; the first notified the availability of the mammography screening program within the Cadiz‐La Janda health district area, specifying the participants' age group from 45 to 69 years, and stating in barely two lines the importance of early diagnosis, without explaining this in any depth nor specifying percentages or numerical values of the benefit of the procedure. There was no mention of possible risks. The notice of an appointment was also attached, indicating the date and time when the woman called should attend for examination. The second page provided a series of instructions on documentation that the woman should bring when attending, and recommendations about appropriate clothing and hygienic aspects to facilitate the procedure. Lastly, the benefit of participating in the program was repeated, although again without quantifying or specifying the magnitude of this benefit.

#### Intervention group

Each woman assigned to the intervention group (IG) received precise verbal and written information on the benefits and risks of the screening program. The intervention was no mere delivery of documentation; the contents of the Cochrane leaflet were verbally explained to the women, as is standard in informed consent procedures. This information was based on the 1st edition (2008) of the document created by the Nordic Cochrane Centre, Copenhagen (Denmark), a Spanish translation of which can be consulted in the following Websites: www.screening.dk and www.cochrane.dk (see Data S1). The leaflet was translated by a native Spanish speaker, revised, and backtranslated verbally with a person to check validity. The document obtained a satisfactory readability score (the Flesh‐Kincaid Reading Ease score was 84.2), and gives quantitative information on the benefits of mammography screening (1 death from breast cancer will be avoided in every 2000 women submitted to screening by mammography in 10 years) and on the risks of the program (10 women in every 2000 will be diagnosed and treated unnecessarily, and in every 200 a false positive result will be produced that will affect the woman psychologically). Additionally, information is offered on other possible harmful effects such as breast pain secondary to the compression of the breasts, the exposure to ionizing radiations, and the sensation of false security.

#### Control group

They received same information as Intervention but did not receive verbal and written information based on the Cochrane leaflet.

In addition, women of both groups were free to request and seek any information whenever they wished. For this they were given another document with a list of sources they could access to find more information (see Data S1).

One month after the first interview the second was carried out, by telephone, with the object of evaluating again their knowledge, attitudes, decision, anxiety/depression and fear of cancer. If the first attempt to contact the participant by telephone was unsuccessful, she was called again, up to a maximum of three times. If finally it was not possible to make contact with the participant, this person was excluded from the study.

Before study commencement the three researchers reached a consensus on a standard form and content of the information to be provided to those in the IG. Adherence to the information protocol during the study was ensured by monitoring the content of the informative document (see Data S1).

Participants with borderline/pathological diagnosis on the Hospital Anxiety and Depression Scale (HADS) were recommended to consult their corresponding mental health professional.

### Objectives

#### Principal objective

The aim of this study was to evaluate the influence of receiving adequate information on the real benefits and risks of mammography on the level of knowledge of participants in the screening program. The secondary objectives of this study was to evaluate how such information might influence the participants' attitude, decision to participate, anxiety/depression, and worries about cancer.

As an additional analysis, the level of knowledge of the different subgroups was studied.

### Outcomes

The questionnaires for measuring the level of knowledge, attitude, and psychosocial impact have been previously described 13. All the questionnaires administered (translated into English) are in supplementary material.

Briefly, the knowledge analysis questionnaire was adapted from a questionnaire developed by the Health Decision Group of the Sydney School of Public Health, Australia (www.health.usyd.edu.au/shdg/resources/decision_aids.php), and was further modified to take into account BCS benefits and risks as described in a 2008 document (available in several languages, including Spanish) developed by the Nordic Cochrane Centre based in Copenhagen, Denmark (www.cochrane.dk). The questionnaire had four multiple‐choice questions, each with three possible answers, scoring 1 point each, and three free‐response questions, each with one possible answer, scoring 2 points each. Minimum and maximum scores were 0 and 10, respectively. Women were considered to be well informed (to have “good knowledge”) for scores of 5 or more. All women were scored, but any questions left blank were not scored 13 (the multiple‐choice and free‐response questions and possible answers are included in the supplementary material).

Attitude was calculated as per Marteau et al. 14 for four questions, each scoring between 0 (most positive) and 6 (most negative), for a maximum score of 24. The questions were as follows: “For me, BCS is (1) a good thing/bad thing, (2) beneficial/harmful, (3) important/not important, and (4) pleasant/unpleasant”. Scores of 13 or more denoted a negative attitude. The internal reliability of the attitude scale was acceptable (alpha coefficient 0.83) 14.

The decision making measures were as follows: “I have decided to participate”, “I have decided not to participate”, and “I am undecided”. They were now being asked whether they would participate again in the future, given the new information.

Mood and cancer worry were measured using the HADS 15 and the Cancer Worry Scale (CWS), 16 respectively.

### Size of the sample

It was estimated that 20% of women in the control group (CG) had good knowledge. To detect a change of 15% in the percentage considered to have good knowledge of the subject, a total of 166 women per group are required. A power of 80%, a level of significance of *P =* 0.05, and a test of two tails have been taken as the statistical criteria. The loss of 20% of the total sample during the study has been assumed. For the interviews, participants were distributed equally among the three researchers.

### Randomization and sequence generation

A random assignment stratified by age, existence of family history or friends with cancer; and educational level, was carried out. Random numbers generated by computer were employed. The two strata established in each category were as follows: women aged 45–59 years/60–67 years; women with/without family history or friends with cancer; and women with secondary or university educational level / with primary level or no formal educational qualification.

The participants were assigned consecutively to each researcher administering the intervention.

### Allocation, concealment, and implementation

The research assistant, who had no contact with the participants, generated the random assignment sequence, and assigned the participants (recruited by the researchers) to one or other group. This method ensured blinding up to the point when interventions were assigned.

### Blinding

The participants and the researchers administering the intervention had knowledge of the group to which each participant had been assigned. However, the research assistant and the statistician of the team were blinded to the assignment, as the randomization of the participants and the data analysis were performed with no knowledge of the group to which any particular participant had been assigned.

### Statistical analysis

For the descriptive analysis of data, absolute and relative frequencies for the qualitative variables, and means and standard deviation for the quantitative variables were calculated. For the comparison between the two groups, the Chi‐squared test was employed for qualitative, and the Student's *t*‐test for quantitative variables.

The Relative Risk of the factors influencing the level of knowledge of participants has been calculated by logistic regression, with a univariate analysis being made of each variable separately. The multivariate model has also been constructed (similarly by logistic regression), introducing all the variables that were previously found to be significant and adjusting for other variables of interest, such as age.

The program used for the statistical analysis is the Statistics Package for the Social Sciences (SPSS, Inc., Chicago, IL, USA) version 12.

## Results

### Participant flow

**Figure 1:**
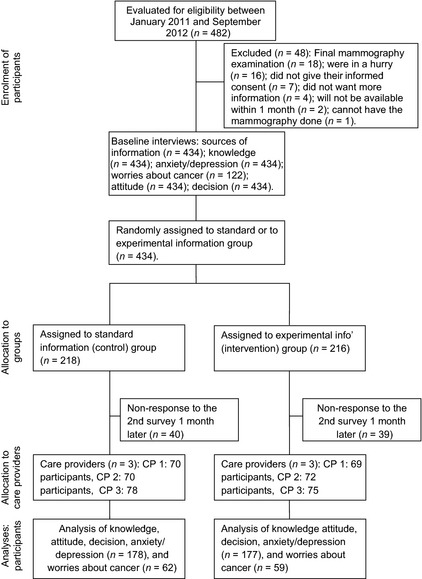
Consort flow diagram.

Figure 1 is the study flow diagram, as recommended in the Consolidated Standards of Reporting Trials (CONSORT).

### Recruitment

Of the 482 potentially eligible participants selected, 434 were finally recruited in 28 visits by researchers to the Program Centre. Almost all visits were made in the afternoon, between 16.00 and 20.00 h. Each visit researchers interviewed a mean total of 17 women. In the second round of interviews, 355 women were interviewed by telephone.

### Baseline data

Table 1 gives the principal characteristics of participants; Figure 1 shows the number of participants attended by each healthcare professional (J. M. B. C., P. R. V., and M. G. G.).

**Table 1:** Basic characteristics of the participants in the control and intervention groups

Characteristics	Subcategory	Control group *N* (%)	Intervention group *N* (%)	*P*‐value
Participants		218 (100)	216 (100)	
Mean age, standard deviation (SD)		54 (6.50)	54 (6.80)	0.350
Range		45–67	45–67	
Family history of breast cancer	Direct	23 (11)	27 (12)	0.240
	Indirect	42 (19)	29 (13)	
Friend or acquaintance with breast cancer		144 (66)	146 (68)	0.760
Personal history of cancer		8 (4)	8 (4)	1.000
Previous false positive in mammography		43 (20)	33 (15)	0.250
Previous participations in screening	None	42 (19)	38 (17)	0.670
	1	39 (18)	32 (15)	
	2	31 (14)	33 (15)	
	3	33 (15)	35 (16)	
	4	21 (10)	20 (9)	
	5	18 (8)	12 (6)	
	More than 5	34 (16)	46 (21)	
Marital status	Married	155 (71)	157 (73)	0.620
	Single	20 (9)	25 (12)	
	Widowed	21 (10)	15 (7)	
	Separated	22 (10)	19 (9)	
Educational level	None	17 (8)	23 (11)	0.760
	Primary	107 (49)	101 (47)	
	Secondary	58 (27)	55 (25)	
	University	36 (16)	37 (17)	
Occupational status	In active employment	75 (34)	75 (35)	0.800
	Housewife	95 (44)	86 (40)	
	Unemployed	30 (14)	33 (15)	
	Pensioner	18 (8)	22 (10)	
Social status^a^	Low	139 (64)	132 (61)	0.310
	High	79 (36)	84 (39)	
Functional capacity (ECOG^b^)	0	189 (87)	179 (83)	0.230
	1	26 (12)	36 (17)	
	2	3 (1)	1 (0.5)	
Associated diseases	Cardiovascular	44 (20)	59 (27)	0.090
	Pulmonary	13 (6)	16 (7)	0.570
	Metabolic	60 (27)	50 (23)	0.320
	Renal	8 (4)	10 (5)	0.390
	Digestive	23 (11)	25 (12)	0.760
	Rheumatological	54 (25)	55 (25)	0.910
	Auto‐immune	8 (4)	7 (3)	1.000
	Psychiatric	32 (15)	16 (7)	0.020
	Hematological	10 (5)	9 (4)	1.000
	AIDS	0 (0)	1 (0.5)	0.490
	Mammary pathology	33 (15)	25 (12)	0.320
Score on the knowledge questionnaire (basal), Mean (SD)		2.97 (1.16)	2.96 (1.23)	0.930
Range		1–7	0–7	
Score on the knowledge questionnaire (gathered at 1 month), Mean (SD)		3.43 (1.39)	3.95 (1.78)	0.002
Range		1–7	1–10	
Score on the attitude questionnaire, Mean (SD)		3.17 (2.69)	3.26 (2.62)	0.720
Range		0–14	0–15	
Score on the *Hospital Anxiety and Depression Scale*, Mean (SD)	Anxiety	1.94 (3.52)	1.78 (3.00)	0.620
Range		0–21	0–13	
Mean (SD)	Depression	0.76 (2.15)	0.69 (1.84)	0.710
Range		0–18	0–14	
Score on the *Cancer Worry Scale*, Mean (SD)		9.92 (3.28)	8.87 (2.69)	0.056
Range		6–23	6–18	
Decision	Decided	218 (100)	216 (100)	1.000

a Socioeconomic level was agreed between the researcher and respondent.

b Eastern Cooperative Oncology Group. ECOG 0: Fully active, able to carry on all predisease performance without restriction. ECOG 1: Restricted in physically strenuous activity but ambulatory and able to carry out work of a light or sedentary nature, for example, light house work, office work. ECOG 2: Ambulatory and capable of all self‐care but unable to carry out any work activities. Up and about more than 50% of waking hours.

### Numbers analyzed

Numbers of women assigned to the standard information (control) and experimental information (intervention) groups were 216 and 218, respectively. Of these totals, 178 of the control and 177 of the IG were included in the analyses of knowledge, anxiety/depression, attitude, and decision. The analysis of worries about cancer was performed with only 62 control and 59 IG participants, because this scale was introduced in the study some time after it had been initiated.

### Outcomes and estimation

In the CG 15 of 178 women (8.40%) acquired a good level of knowledge in the second interview, whereas in the IG 32 of 177 (18.10%) acquired a good level (*P *= 0.008). The mean score obtained in the questionnaire on knowledge by women in the CG increased from 2.97 (SD 1.16) in the first interview to 3.43 (SD 1.39) in the second; the mean score of those in the intervention information group increased from 2.96 (SD 1.23) to 3.95 (SD 1.78); this difference was statistically significant (*P *= 0.002). The women assigned to the IG have a relative risk of acquiring a good level of knowledge 2.39 times greater than those of the CG (95% confidence interval [CI] 1.24–4.60).

After the intervention, 176 participants of the CG (98.9%) presented a positive attitude to the screening program, with only 2 (1.10%) being negative. Similarly, in the IG 176 women (99.40%) presented a positive attitude, and only 1 (0.60%) negative (*P = *1.000). The result of the survey on attitude to the program after the intervention was a mean score of 3.20 (SD 2.81) in the CG, compared with 3.08 (SD 2.64) in the IG (*P = *0.680).

After the intervention, in the CG 178 women (100%) were decided in favor of participating, and none was undecided. In the IG 175 women (98.90%) were decided in favor of participating, and 2 women (1.10%) were undecided (*P =* 0.240).

In Table 2 it can be seen that, after the intervention, the distribution of the participants according to diagnostic category from the range of scores obtained in the subscales of the HADS showed no difference between the two groups. The mean score on the survey items that evaluate anxiety and depression, after the intervention, is presented in Table 2; again there are no differences between the two groups. The survey of worries about cancer also presented scores with no difference between the groups (Table 2).

**Table 2:** The Hospital Anxiety and Depression Scale and worries about cancer questionnaires after the intervention

Category	Subcategory	Control group *N* (%)	Intervention group *N* (%)	*P* value
Anxiety	Normal	161 (90)	164 (93)	0.270
	Borderline	6 (3)	8 (4)	
	Pathological	11 (6)	5 (3)	
Anxiety Mean score (SD) Range		2.02 (3.70)	1.65 (3.00)	0.300
		0–21	0–15	
Depression	Normal	173 (97)	173 (98)	0.840
	Borderline	3 (2)	3 (2)	
	Pathological	2 (1)	1 (0.6)	
Depression, Mean score (SD)		0.71 (2.20)	0.73 (1.96)	0.920
Range		0–18	0–14	
Worries about cancer, Mean score (SD)		9.92 (3.28)	8.85 (2.69)	0.053
Range		6–23	6–18	

### Ancillary analyses

Participants with immediate family members affected by breast cancer acquired a higher level of knowledge than those with more distant family members affected. Participants with a higher educational level (secondary and university) also acquired a higher level of knowledge than those with a lower educational level (primary or none). Similarly participants in active employment, unemployed or pensioners acquired more knowledge than housewives (Table 3). In the multivariate model, introducing the significant variables and adjusting for age as continuous variable, the type of intervention and the educational level maintain their influence on the level of knowledge acquired after the intervention (Table 3). In the subgroup analysis most women improved their level of knowledge.

**Table 3:** Relative risks (RRs) of the various subgroups with respect to the “level of knowledge” variable, univariate, and multivariate analysis

Variables	Subcategory	Univariate analysis		Multivariate analysis	
		RR	95% CI	RR	95% CI
Study group	Control	1		1	
	Intervention	2.39	(1.24–4.60)	2.57	(1.32–5.00)
Age	45–59	1.85	(0.83–4.13)		
	60–69	1			
Family history with breast cancer	None	1			
	Immediate (first degree) family members	1.66	(0.71–3.93)		
	Other family members	0.42	(0.14–1.24)		
Friends or acquaintances with breast cancer	Yes	0.96	(0.49–1.85)		
	No	1			
Personal history of cancer, other than breast cancer	Yes	0.99	(0.21–4.54)		
	No	1			
Previous false positive mammogram	Yes	0.82	(0.35–1.93)		
	No	1			
Previous participations in screening	None	1			
	1 or more	0.89	(0.41–1.96)		
Marital status	Married	1			
	Single, Widowed, Separated	1.68	(0.88–3.19)		
Educational level	None, Primary	1		1	
	Secondary, University	2.32	(1.23–4.37)	2.05	(1.01–4.17)
Occupational status	Housewife	1		1	
	Active, Unemployed, Pensioner	2.45	(1.20–5.00)	2.03	(0.93–4.38)
Social status	High	1.29	(0.69–2.41)		
	Low	1			

### Adverse events

The participants with pathological or borderline diagnosis on the HADS presented no differences between IG and CG (Table 2).

## Discussion

Our study evaluates the effectiveness of a model of informed consent on mammography screening for early detection of breast cancer; the model tested meets all the requirements of an informed consent. This intervention was found to improve considerably the level of knowledge of the participants (8.40% vs. 18.10% acquired a level of knowledge rated as good), but was insufficient to consider that the women receiving the information had taken an informed decision (their mean score was 3.95 of 10, whereas the minimum acceptable score was 5 of 10). Nor did the intervention modify their attitude or decision (positive for the great majority) on submitting to mammography or their degree of anxiety, depression, and worries about cancer.

Other studies have also evaluated the effectiveness of different informative materials in improving the knowledge of participants in mammography screening [7, 17, 18, 19, 20]; these demonstrate that, in general, such information increases the level of knowledge and serves to help these women take a more informed decision. As in our study, these other studies also find that these informative interventions do not modify the attitude toward the screening, nor do they make the women more or less anxious, depressed, or worried about cancer [17, 18].

The basic document used for the informed consent in our study was the informative leaflet drafted by the Nordic Cochrane Centre 21. It was chosen because it represents very complete informative material; it is objective and clear; and it includes data based on the evidence regarding the benefits and dangers, presented in absolute terms. It is available in Spanish, and when properly assimilated, it does enable the woman to take a well‐informed decision. However, although the intervention constituted an adequate communication of the information for enabling informed decision making 22, it was not very efficacious in assisting the women in our sample actually to make their decision. The women tended to have an exaggerated view of the benefit from the screening program, and did not understand the concept of overdiagnosis 13. Other authors have also questioned why women have difficulties in understanding the concepts of early diagnosis of breast cancer, overdiagnosis and false positives [23, 24]. Therefore the key question is not what information should be given to the women 24, but rather, how to make sure that the information transmitted is understood and assimilated, and how to verify this 25. There is the possibility that women in the IG were indeed making a more well‐informed decision, but that the additional knowledge did not impact the decision for screening. In other words, close to 100% of women would still undergo screening even despite understanding the risks of overdiagnosis and overtreatment.

It is possible that, as was done in our study, including an appropriate healthcare professional in the mammography screening procedure to obtain an individual informed consent in the conventional way, may not be the most effective option for communicating. However, this model for providing, or at least offering, information, by the professionals, such as those of primary care, as a basic measure before the woman's first screening by mammography, could be interesting [26, 5].

Despite these findings, the authors are pessimistic regarding the majority of women actually making a properly informed choice. Public opinion now seems to be solidly in favor of such screening; there is almost universal social acceptance that it is “a good thing”, based on widespread confidence in public health institutions and gratitude to them for offering free screening, reinforced by the paternalism underlying social behavior today [25, 27, 28]. When the mass communications media repeatedly issue persuasive messages promoting mammography with no qualification or facts on known risks and dangers, this serves to strongly reinforce the sense that there is no need to raise questions or express fears, that is, to be properly informed as an individual.

The routine way to provide information and obtain informed consent for medical interventions is to provide verbal information in a personal interview and written information that patients can read in light of their own values. This will in theory help patients increase their knowledge, modify (or not) their attitude and support (or not) their decision to undergo the medical intervention. This routine action performed daily by doctors is what we wanted to test in the context of breast screening. If an individual paper‐based intervention can support patients in making informed decisions about treatments, why not in the context of secondary prevention of cancer via breast screening? This is what we have tried to respond to in this trial. It is clear in our study that this educational intervention is of limited use. Recommendations based on lessons learned as applied to the design of interventions for women undergoing mammography should – as well as including an informed consent as tested in our trial – also include an aid to decision making that – transparently and objectively – describes all the benefits and risks of BCS 29 while also respecting the autonomy of the women. Rather than use the percentage of participation in screening it would be preferable to use the agreement between women's preferences and their degree of participation 30. Moreover, interventions should also be implemented to train health professionals, revising university curriculums and ensuring that continuing medical education includes training in risk literacy and communication 31. Finally, the general population (not just women at risk of breast cancer) should also be informed, for instance, through early school‐based interventions aimed at children and teenagers 31.

The strengths of our study include its randomized design, similar characteristics across groups, enough participants to meet the study goal, and pre and postinformation obtained for each participant. On the limitations of this clinical trial, the women who received the experimental information may have perceived it as generally arguing against mammography, being accustomed to receiving information that only emphasizes the benefits, and aimed at maximizing participation [22, 5]. In addition, implementation of the study immediately after screening of women with a positive attitude who had recently made their decision may undermine the effectiveness of the intervention, especially in terms of changing both attitude and decision. People seek consonant and avoid dissonant information 32. Regarding external validity, it may not be valid to generalize our findings, given the differences in detail in the information received by women in the screening programs of different countries, regions and even different health districts of the same region. Other possibly limiting factors include the level of education and of awareness of the health variables according to the population 33. The transfer of the experimental information provided to participants early in the study to other women called for screening at later dates is another possible source of bias, since social connections (e.g., between neighbors and acquaintances) may have existed between the women sampled in our study.

## Conflict of Interest

J. N. V. worked as epidemiologist in the Screening Program in the previous 3 years; no other relationships or activities that could appear to have influenced the submitted work.

## Supporting information

Data S1. Study protocol.
