# Supplementary material for: Using an informed consent in mammography screening: a randomized trial
Source: Cancer Med. 2015 Sep 17;4(12):1923–32. doi: 10.1002/cam4.525 (PMC5123785; doi:10.1002/cam4.525)
Supplement: Supplementary file 1 — Data S1. Study protocol. [file CAM4-4-1923-s001.doc]

**Element included in the document:**

1. Protocol

2. Annex 1. Official information by letter

3. Annex 2. Experimental information

4. Annex 3. Sources that you can access to request information

5. Annex 4. Questions on the program of screening by mammography and method of assessment (i.e. Questions about level of knowledge of participants)

6. Annex 5. Questionnaire on the sources of information

7. Annex 6.Assessment of the attitude towards the program of screening by mammography

8. Annex 7. Questionnaire on the decision to participate in the mammography screening

9. Annex 8. Questionnaire for assessment of the anxiety and depression related to the information on mammography provided

10. Annex 9. Questionnaire for assessment of worries about cancer

**Appendix 1. Protocol**

**SUBSIDIES FOR THE FINANCING OF BIOMEDICAL AND HEALTH SCIENCES RESEARCH IN ANDALUSIA**

**RESEARCH PROJECTS**

**TECHNICAL-SCIENTIFIC AND ECONOMIC REPORT**

Order of 1 June 2010 (BOJA Nº 114 dated 11/06/2010)

| PRINCIPAL RESEARCHER | |
| --- | --- |
| **FAMILY NAMES**  Baena Cañada | FIRST **NAME**  José Manuel |

| TITLE OF THE PROJECT |
| --- |
| **Randomised clinical assay on the effectiveness of a model of informed consent based on an understanding of the benefits and risks in women submitted to population screening by mammography** |

| KEY WORDS |
| --- |
| Autonomy, capture, screening, ethics, information, mammography |

| SUMMARY |
| --- |
| (Maximum 250 words)  Objectives: to evaluate the effects attributable to receiving adequate information on the real benefits and risks of mammographies, in women who participate in the population screening program, in terms of level of knowledge, attitudes, intention with respect to the screening, fear of cancer, anxiety and conflict about the decision. Design: controlled clinical assay, with random assignment, of non-pharmacological intervention. Subjects and scope of study: women called to have a mammography under the population screening program in the Bay of Cadiz-La Janda Health District. Instrumentalization: random assignment of the participants in two subgroups in accordance with the explanatory information provided. The control subgroup is that of standard information, which consists of receiving the information customarily received by the women invited to attend the program for the early detection of breast cancer. The experimental subgroup consists of those receiving from a researcher accurate factual information, verbal and written, on the real benefits and risks of the mass screening programs by mammography. Determinations: knowledge, attitudes, intention with respect to the screening, fear of cancer, anxiety, and conflict about the decision. |

| **1. SCIENTIFIC-TECHNICAL ASPECTS OF THE PROJECT** |
| --- |

| 1.1 | BIBLIOGRAPHY |
| --- | --- |
| It is important that the bibliography should be up-to-date and pertinent to the topic proposed. Citation of the bibliographic references over the course of the project. (Maximum 2 pages)  1. World Health Organization Web site. (Accessed November 24, 2009, at http://www.who.int/mediacentre/factsheets/fs297/en/index.html.)  2. Nyström L . Long-term effects of mammography screening: updated overview of the Swedish randomised trials. Lancet 2002; 359 (9310): 909 – 919.  3. Gøtzsche PC, Nielsen M. Screening for breast cancer with mammography. Cochrane Database Syst Rev. 2006; 4 : CD001877.  4. Humphrey LL, Helfand M, Chan BK, Woolf SH. Breast cancer screening: a summary of the evidence for the U.S. Preventive Services Task Force. Ann Intern Med 2002; 137(5 Part 1):347–60.  5. Nelson HD, Tyne K, Naik A, Bougatsos C, Chan BK, Humphrey L. Screening for Breast Cancer: An Update for the U.S. Preventive Services Task Force. Ann Intern Med 2009;151:727-737.  6. John EM, Phipps AI, Knight JA, Milne RL, Dite GS, Hopper JL, et al. Medical radiation exposure and breast cancer risk: findings from the Breast Cancer Family Registry. Int J Cancer. 2007;121:386-94.  7. Armstrong K, Moye E, Williams S, Berlin JA, Reynolds EE. Screening mammography in women 40 to 49 years of age: a systematic review for the American College of Physicians. Ann Intern Med 2007;146:516-26.  8. Brett J, Bankhead C, Henderson B, Watson E, Austoker J. The psychological impact of mammographic screening. A systematic review. Psycho-oncology 2005;14:917-38.  9. Brewer NT, Salz T, Lillie SE. Systematic review: the long-term effects of false-positive mammograms. Ann Intern Med 2007;146:502-10.  10. Mushlin AI, Kouides RW, Shapiro DE. Estimating the accuracy of screening mammography: a meta-analysis. Am J Prev Med. 1998;14:143-53.  11. Hofvind S, Thoresen S, Tretli S. The cumulative risk of a false-positive recall in the Norwegian Breast Cancer Screening Program. Cancer. 2004;101:1501-7.  12. Elmore JG, Barton MB, Moceri VM, Polk S, Arena PJ, Fletcher SW. Ten-year risk of false positive screening mammograms and clinical breast examinations. N Engl J Med. 1998;338:1089-96.   1. 13. Moss S. Overdiagnosis and overtreatment of breast cancer: overdiagnosis in randomised controlled trials of breast cancer screening. Breast Cancer Res. 2005; 7:230-4.   14. Castells X, Sala M, Ascunce N, Salas D, Zubizarreta R, Casamitjana M, coordinadores. Descripción del cribado del cáncer en España. Proyecto DESCRIC. Madrid: Plan de Calidad para el Sistema Nacional de Salud. Ministerio de Sanidad y Consumo. Agència d’Avaluació de Tecnologia i Recerca Mèdiques de Cataluña; 2007. Informes de Evaluación de Tecnologías Sanitarias, AATRM núm. 2006/01.  15. Webster P, Austoker J. Does the English Breast Screening Programme’s information leaflet improve women’s knowledge about mammography screening? A before and after questionnaire survey. Journal of Public Health 2007; 29 (2): 173–177.  16. Raffle AE. Information about screening - is it to achieve high uptake or to ensure informed choice?. Health Expectations 2001; 4:92-98.  17. Mathieu E, Barratt A, Davey HM, McGeechan K, Howard K, Houssami N. Informed Choice in Mammography Screening. A Randomized Trial of a Decision Aid for 70-Year-Old Women Arch Intern Med. 2007;167(19):2039-2046.  18. Mathieu E, Barratt A,. McGeechan K, Davey HM, Howard K, Houssami N. Helping women make choices about mammography screening: An online randomized trial of a decision aid for 40-year-old women. Patient Educ Couns (2010), doi:10.1016/j.pec.2010.01.001.  19. Jepson RG, Forbes CA, Sowden AJ, Lewis RA. Increasing informed uptake and non-uptake of screening: evidence from a systematic review. 2001 Health Expectations 2001; 4: 116-130.  20. Gigerenzer G, Mata J, Frank R. Public knowledge of benefits of breast and prostate cancer screening in Europe. J Natl Cancer Inst 2009;101:1216–1220.  21. Jørgensen KJ, Gøtzsche PC. Overdiagnosis and overtreatment. Content of invitations for publicly funded screening mammography. BMJ 2006; 332:538-41.  22. Gøtzsche PC, Hartling OJ, Nielsen M, Jørgensen KJ, Brodersen J. Breast Screening - The Facts. Or maybe not. BMJ 2009; 338:446-8. | |

| 1.2 | ANTECEDENTS AND CURRENT STATE OF THE STUDY TOPIC |
| --- | --- |
| Knowledge on the antecedents and current state of the topic will be valued (Maximum 3 pages)  Breast cancer is the most frequent malignant neoplasia in woman globally. It is the tumour that causes the most deaths in women in our country. Thus breast cancer represents both a healthcare and a social challenge, and justifies the need to promote and conduct new studies to improve the health of these patients. During the two last decades there has been a modest but real fall in mortality due to breast cancer, and this has been attributed to improvements in early detection and treatment (1).  Mammography is currently the best technique for population screening for breast cancer, and there is a general consensus that such screening produces a reduction in mortality. Nevertheless, it is also widely recognized that mammography is an imperfect diagnostic test that fails to detect a considerable number of cancers, particularly those that are hormone resistant - cancers that represent the greatest threat to the survival of women. Mammography also detects a considerable number of false positive cases, and over-diagnoses cancers that would never have had serious clinical consequences, particularly non-invasive and some invasive tumours.  Everyone agrees that population screening by mammography saves lives, so early diagnosis programs are run in all the healthcare systems of developed countries. A meta-analysis of the randomised trials in which 247,000 women aged between 40 and 74 years were recruited, with a follow-up of between 5.8 and 20.2 years, has shown that for every 1000 women participants in the screening, 3.9 died from breast cancer, compared with 5 deaths from breast cancer among non-participants (2). The reduction of the absolute risk was of the order of 1 in 1000 women. Another review of 6 clinical trials in which half a million women participated reported a reduction of 15% in mortality, with a reduction of the absolute risk of 1 in 2000 (3). A review of the preventive services of the United States found similar effects in a meta-analysis of 7 clinical trials: a reduction in mortality of 16% and an absolute benefit of 1 death prevented per 2000 women screened (4). Recently this group has published a new review specifying the benefit obtained by women depending on their age (5). The absolute benefits by age group are: 1904 women submitted to mammography in order to save 1 life in those aged from 39 to 49 years; 1339 women in those aged from 50 to 59 years; and 377 women in those aged from 60 to 69 years (5).  However it continues to be a topic of debate whether the potential of the screening programs to reduce mortality from breast cancer exceeds the potential harm caused by them. The radiation emitted during the exposure of the breasts in a mammography is considered a low dose, of low energy. Although the exposure to a high dose is known to be related to an increased risk of breast cancer, the relationship with the exposure to a low dose is inconsistent (6).  The pain and mammary discomfort secondary to the glandular compression while the mammography images are obtained affects each woman differently, to a very variable degree. Between 1 and 77% of women make reference to it, although very few attach sufficient importance to it to dissuade them from participating in the screening program (7).  The anxiety associated with the mammography screening is usually transitory and of little significance, although in some cases it can be persistent (8). The false positive findings do not seem to provoke negative effects in the form of anxiety and depression in general for the women participants, but in some cases they do provoke anxiety, apprehension and a sensation of risk specifically associated with the breast cancer (9).  Accurate data on false positive and negative mammography results, on the need for additional images to be studied and for biopsies, are limited. More than 10 years ago the rate of false positives was estimated at between 0.9 and 6.5% (10). The cumulative figure after a woman has undergone 10 mammographies reaches rates of 21 to 49% (11) and up to 56% in the case of young women (12).  The over-diagnosis of cancer is another potentially harmful effect of screening programs. A meta-analysis that analyzed this subject estimates that the excess number of invasive and in situ cancers over-diagnosed is between 0.07 and 0.73 cases per 1000 women and year (13). This means that some women are diagnosed with breast cancer, but this diagnosis would never have been made if the woman had not participated in the screening. These women will also undergo a treatment that they would never have undergone without their participation. The Cochrane review highlighted that the breast was extirpated in 20% more women in the screening group than in the control group (3).  It is a mistake to assume that the scientific data can tell a person what is the correct decision with respect to a medical choice. Individuals should take their own decisions regarding medical treatments and other interventions, basing the decisions on scientific data, but each person must evaluate the possible results according to their own beliefs and values. This applies not only for topics of special significance such as abortion or withdrawal of life support in a terminal disease, but also for every medical choice, no matter how trivial that may be. Although there may be variability between regions (14), at least in our geographic area, in the case of population screening by mammography, these assertions are not currently being met, since women do not have access to accurate scientific information on the potential benefits and harm nor can they, therefore, take the decision in accordance with their own values based on such information. In our area, the health systems, scientific societies and communications media exert influence over women following the obsolete principle of authority, effectively keeping the woman herself marginalised in the decision on whether or not to be submitted to screening by mammography. Women can obtain some information on the mammography screening programs, but this is not optimum (15), and such information is directed more towards achieving a high rate of participation than to ensuring that an informed choice is made (16). The improvement in the information that is provides to a woman called for screening by mammography should originate from a healthcare professional with the correct training. During the process of informing, the values of the doctor and the woman should coincide, although in reality the values of the doctor should not count. The woman's fears and anxieties may condition her decision. If the doctors have systematic biases on how to weigh the benefits against the risks, these will be reflected in their recommendations.  It has been demonstrated that help in taking decisions by providing balanced quantitative information on the screening programs with mammographies increases the women's knowledge and is a way of assisting them in their personal decision (17). The improvement of the information on benefits and harms of mammographic screening reduces the indecision of the women without increasing their anxiety (18). It is not clear if the informed choice of the women increases or reduces their participation in the screening program, although there is some evidence that improving the interventions aimed at informing women does not alter their decision (19). In this context, new and better designed clinical trials are necessary directed towards clarifying the true impact of the information on women; it is also essential to develop and validate instruments of accurate information to ensure that the woman is fully informed and able to decide if mammographic screening is the correct choice for her (19).  The official information received by women called for screening by mammography does not include data about the real benefits, nor about the risks of over-diagnosis or over-treatment, and the palpable reality is that the level of information that these women have been shown to possess is very deficient (20)(21). For this reason, models of information have been designed, such as that of the Danish group, which has been translated into Spanish, which may be applicable in different healthcare contexts (22). | |

| 1.3 | RESEARCH HYPOTHESIS OR QUESTION |
| --- | --- |
| The clarity and specificity of the research hypothesis or question will be valued.   - - Accurate information on the benefits and risks of the population screening program by mammography increases the knowledge of women about this subject.   - Accurate information on the benefits and risks of the population screening program by mammography allows the women participants to make an adequate informed decision on whether to continue or to stop submitting themselves to this procedure.   - Accurate information on the benefits and risks of the population screening program by mammography modifies the level of actual participation of these women.   - Women provided with accurate information on the benefits and risks of the population screening program by mammography do not suffer increased anxiety and fear of cancer. | |

| 1.4 | OBJECTIVES |
| --- | --- |
| Enumerate briefly, but clearly, precisely and realistically (that is, according to the forecast duration of the project) the specific objectives that are sought. The clarity, relevance and novelty of the objectives will be valued.  Principal objective:   - To evaluate how adequate information on the real benefits and risks of mammography influences the women participants in the screening program, in terms of knowledge, attitudes, intention of participating, fear and anxiety.   Secondary objectives:   - To measure the level of knowledge of the women on the population screening program by mammography. - To analyse the attitudes and the decision taken by the women after providing them with accurate information on the benefits and risks of the population screening program by mammography, on whether to continue or stop being submitted to screening by mammography, and whether this decision results in effective participation. - To analyse what are the principal sources of the information that the women possess on the benefits and risks of the screening programs. - To measure the degree of anxiety and fear of cancer in the women called for population screening by mammography. | |

| 1.5 | METHODOLOGY |
| --- | --- |
| Detail and justify the activities or tasks that are to be carried out. The viability of the research project will be valued: appropriateness for the objectives of the methodology, the design of the research, analysis of the data, and plan of work (maximum 5 pages).  Study design:  This is a controlled clinical assay of random assignment, of non-pharmacological intervention. The women will be assigned to one of the two groups according to the information each receives on the benefits and risks of the program of screening by mammography, by means of simple random assignment using a system that generates random numbers. Stratification will be applied in the sampling, preserving in both groups similar percentages of age group, educational level, family antecedents of breast cancer, and friends or acquaintances with breast cancer.  The group taken as the control is that comprised of women receiving the standard information; this is the information that the public institutions provide to all the women called for the program for early diagnosis of breast cancer (See Annex 1). The intervention group is that comprised of women receiving the experimental information; this is accurate information received verbally and in writing on the benefits and risks of the mammography screening program, provided by the research team. Both groups of women were also able to request and seek information, as they wished, either through the healthcare professionals, or through the communications media, from associations of patients, from medical publications or informative leaflets, or from the Internet. The recruitment of the women and the intervention to be made will be carried out when they attend for performance of the mammography screening in the centre of the Bay of Cadiz-La Janda District Health (Avenida Ramón de Carranza 19, Cadiz). The interview with the researchers will take place after the performance of the mammographic study, so as not to interfere with the decision previously taken by the woman. In this interview the informed consent for participation in the study will be obtained; then the level of knowledge, sources of information, attitudes, anxiety and fear of cancer will be analysed in both groups of women. A month after the intervention a telephone interview will be carried out to analyse again the knowledge, sources of information, attitudes, intention with respect to the screening, anxiety and fear of cancer, and decision conflict. Two years later, the women will be called for the next mammography examination. At that time the actual decision taken by the woman will be investigated by telephone interview.  The information that will be provided to the women regarding the benefits and risks of the program of screening by mammography is based on the document devised by the Nordic Cochrane Centre of Copenhagen (Denmark), of which there exists a version translated into Spanish, available on the following Web pages: www.screening.dk and www.cochrane.dk (see Annex 2). The document gives quantitative information on this benefits of mammography screening (1 death from breast cancer will be avoided in every 2000 women submitted to screening by mammography over 10 years). Quantitative information is also provided on the risks of the program (10 women in every 2000 will be diagnosed and treated unnecessarily and in every 200 one false positive result will be produced that will have a psychological impact on the woman). The message we will attempt to transmit is that it would be equally reasonable to participate or not to participate, since it has been demonstrated that the screening has both negative and positive effects, and the objective is that the woman should be given the capacity to make an informed choice. Both groups of women will be able also to request and seek information as and when they may wish. For this they will be given another document with a list of sources which they can access to find more information (see Annex 3).  Case definition. Study subjects:  Women called for the program of early diagnosis of breast cancer, operated by the Bay of Cadiz-La Janda Health District, and who attend for the performance of the mammography. In our health-care area a mammography is offered every two years to women aged between 45 and 69 years, who are invited to participate in a personal letter sent to them by mail.  Inclusion criteria:  1. Women resident in the Bay of Cadiz-La Janda Health District.  2. Women aged between 45 and 69 years.  3. Women called for the mammography screening program and who actually attend.  4. Women who have the capacity to give their informed consent for participating in the study.  Exclusion criteria:  1. Women with antecedents of breast cancer  Criteria for withdrawal of the study:  1. Explicit wish of the patient to leave the study.  Variables:  Principal:   - Level of knowledge obtained in the questionnaire to assess the level of knowledge on the benefits and risks of the mammography screening (see Annex 4). An assessment will be made using questionnaires specifically designed to measure the variable. These questionnaires have been adapted to the informative document that will be administered to the women in this study, from originals developed by the Sydney Health Decision Group of the School of Public Health of Sydney, Australia; these are available on the following web page: http://www.health.usyd.edu.au/shdg/ (see Annex 4). In the questionnaire there are 7 questions, of which 3 are quantitative without assumptions and that are scored with 2 points each. There are also 4 qualitative questions with 3 assumptions, which are scored with 1 point each. The maximum score that a participant can obtain is 10 and the minimum 0. It will be considered that the woman has made a choice based on adequate knowledge if a score higher than 5 is obtained.   Secondary:   - Each of the items of the questionnaire on the sources of information (see Annex 5).The source of the knowledge possessed on the program of screening by mammography will be investigated in all the women included in the study. The women will be asked to estimate the frequency with which they have obtained information from family and friends, from experts (primary care doctor, pharmacist), from the communications media (television, radio, press) and from sources related to the healthcare services or institutions (leaflets and propaganda from the Servicio Andaluz de Salud and from the Ministry of Health), from insurance companies, consumers and patients associations, books, and Web pages on health and medicine in the Internet. The possible responses will be: Never, Rarely, Sometimes, Frequently and Don't know (see Annex 5). - Attitude towards the mammography screening (see Annex 6). The attitude of the women towards the screening mammographic, whether positive or negative, will be investigated. For this they will be asked to respond to 4 questions that will each be scored from 0 to 6. The questions will be formulated as statements with options, as follows: “For me, the screening by mammography is a good option/a bad option, beneficial/harmful, important/not at all important, and pleasant/unpleasant”. The minimum score will be 0 and the maximum, which denotes the most positive attitude towards screening, will be 24. The limit between the positive and negative attitudes is set at halfway on the scale, with a score above 12 being taken to denote a negative attitude towards the mammography screening (see Annex 6). - The type of decision of the woman according to the decision questionnaire (see annex 7). The women will be classified according to their intention with respect to the screening and their final decision (capture). A woman with adequate knowledge and with a positive attitude towards the screening should have the intention to participate in the screening and, finally, should actually participate. A woman with adequate knowledge and with a negative attitude towards the screening should have the intention not to participate and, finally, should not respond to the call to attend the screening. The optional statements that will be put to the women in relation to their decision regarding participation in the mammography screening will be: “I have decided to participate”, “I have decided not to participate”, and “I am undecided” (see Annex 7). - Effective participation in 2 years. - The level of anxiety, feelings (state of mind) and fear of cancer, according to the corresponding questionnaire (see annexes 8 and 9). The anxiety and feelings/state of mind will be measured using the Hospital Anxiety and Depression Scale (HADS) (Zigmond AS, Snaith RP. The Hospital Anxiety and Depression Scale. Acta Psychiatr Scand 1983; 67: 361-70). When the recruitment of the women was already advanced, another specific questionnaire was introduced, to measure worries about cancer, using the Cancer Worry Scale (CWS) (Cabrera E, Zabalegui A, White I.) Spanish version of the Cancer Worry Scale (Escala de Preocupación por el Cáncer: adaptación cultural y análisis de la validez y la fiabilidad). Med Clin (Barc) 2011; 136 (1): 8–12) (see Annexes 8 and 9). - Identification data in respect of each woman. - Age - Comorbidity. - Educational level. The women will be classified in the following 4 categories: “no certified education”, “primary schooling (Educación General Basica (EGB) or similar)”, “secondary schooling (Formación Profesional/Bachillerato or similar)” and “higher studies (University)”. - Social status. The category applied will be: “low”, “medium-low”, “medium-high” or “high”, as agreed between the woman and the researcher. - Occupational status. The category applied will be: “unemployed”, “pensioner”, “housewife” or “in active employment”, as reported by the woman. - Rural or urban domicile. - Family antecedents of breast cancer. - Friends or acquaintances with breast cancer. - Number of previous participations in screening. - Previous false positive.   Collection and analysis of the data:  The data will be collected in data collection notebooks designed for this purpose. The first interview with the women will take place in person at the Health District centre, where the mammography is performed. The second interview about a month later, and the third after 2 years, will take place by telephone.  Size of the sample: Based on a preliminary estimation that 20% of the women in the control group have an adequate level of knowledge, a total of 166 women per group will be required to detect a change of 15% in the proportion of women considered to possess a good level of knowledge. A power of 80%, a level of significance of p=0.05 and a test of two tails will be considered. We also assume that 20% of the original sample will be lost during the study.  Statistical analysis: The analysis will be made in the population by intention to treat, which is defined as the population of all the randomised patients, analysed in the arm to which they have been assigned. The Chi-squared test with Fisher's correction will be applied, if necessary, to compare the differences between the qualitative variables, and the Student-t test for quantitative variables, between the two groups (i.e. the mean score on the level of knowledge between the control and experimental groups). The Student t test will be applied for paired data for the comparison of quantitative variables in the same group over the course of time. Logistic regression will be used to study the association between knowledge and participation, and between attitude and participation, controlling for sociodemographic variables. The program SPSS version 12 will be used for the statistical analysis of the data. Statistical significance will be indicated when p<0.05.  Limitations of the study:  It is possible that the women who participate in this clinical assay will consider the experimental information provided as being contrary to the mammography screening, since they will be accustomed to receiving information that emphasizes only the benefits, and aimed only at achieving a high rate of participation (a high capture). We have designed this clinical assay pragmatically, and appropriate to the local context, where the program of mammography screening does not provide specific and quantitative information on benefits and risks. Regarding the external validity of our study, it may not be possible to generalize our findings, given the particular features of the information received by women in the mammography screening programs of different countries, regions and even the different health areas of the same region. Other possibly limiting factors include the level of education and of awareness of the health variables according to the population. | |

| 1.6 | PLAN OF WORK |
| --- | --- |
| A good description of the plan of work, indicating the person/s who are going to carry out each task, will be valued (maximum 1 page).  This is a clinical assay, based on an original idea of the principal researcher. It will be conducted in collaboration with the Medical Oncology service of the 'Puerta del Mar' University Hospital of Cadiz, the Bay of Cadiz-La Janda Health District and the Population Register of Cancer of Cadiz. It is a population-based study in which women consecutively called for screening will be included. The date foreseen for the start will be January 2011 and the period of recruitment will end December 2011. The epidemiologist, who is one of the collaborating researchers, will be advising on the methodology and statistical aspects of the study. Another epidemiologist, also a collaborating researcher, will be responsible for the coordination and facilities in the screening program centre. The oncologists and residents of Medical Oncology, who are the principal and collaborating researchers, will be responsible for the identification of the cases meeting the criteria for inclusion in the study, and this will be performed actively by their physical presence and the help of the support personnel to the research activity in the centre of the mammography screening program outside their normal hours of work. The support personnel (nurse and research assistant) will attend to all the visits made by the women to the centre of the screening program, and subsequently will be responsible for the telephone interviews one month later, and 2 years later. They will also be charged with the transcription of the data to a computer database, and with coordination with the epidemiologists.  Interview script used by the interviewers:  First interview (baseline):   1. Information on the study 2. Written informed consent 3. Personal details 4. Questionnaire on knowledge, attitude, decision, anxiety, depression and cancer concerns 5. Randomization    1. Control group: document with information sources on BCS    2. Intervention group: verbal and written information on the benefits and risks of the screening programme (Cochrane leaflet) document with information sources on BCS   Second interview (after one month):   1. Questionnaire on knowledge, attitude, decision, anxiety, depression and cancer concerns   The schedule of the study is the following:  Study design: year 2010.  Presentation in Ethic Committee for Clinical Research: April 2010.  Application of financing for the study: June 2010.  Start of the study: January 2011.  Recruitment of the patients and personal interviews: January 2011 to December 2011.  Second interview: February 2011 to January 2012.  Third interview: January 2013 to December 2013.  Analysis of the data and publication / communication of results: year 2014. | |

| 1.7 | ETHICAL ASPECTS OF THE RESEARCH |
| --- | --- |
| The ethical aspects that must be taken into account in carrying out the project will be assessed.  The real situation, which represents the point of departure, is the following: in all health systems, emphasis is given to the importance of the information that must be provided to the users for the clinical decision-making, giving priority to the principle of the user's autonomy; however, the secondary prevention of breast cancer by population mammography screening has developed in a context where the principle of authority prevails. That is, the woman is invited to participate because the health system proposes that it is for her benefit, but without giving her accurate data on those benefits and without presenting the risks. The object is to capture the highest possible proportion of those considered at risk. The ethics of this clinical approach in which the women are not informed is doubtful, to say the least. In this context, the introduction of improvements in the information given to the woman containing accurate data on the benefits and risks, will have a definite clinical impact and be of great healthcare value because it will be the woman herself who, after being capacitated to decide, will take her own decision based on both her own knowledge and her own values. It is to be seen if the improvement in the information is going to lead to a decrease in the rate of attendance (i.e. the capture), but, if it does, this should not be a cause for ethical concern, since the woman will have decided freely.  The women participants in this study will not be submitted to any supplementary diagnostic test, nor to any treatment different from that normally given. The participation will be voluntary and will not represent any cost to the participant. If the woman decides to participate and later changes her mind, she is free to do so, and does not have to give any kind of explanation for withdrawing. The medical care that she will receive will not be affected by her decision. The data of personal character will be treated according to the provisions in the ruling Spanish regulations (Organic Law 15/1999, of 13 December, for the Protection of Data of Personal Character).  The present study does not require insurance, for two reasons: it is not a clinical trial with medication; hence the RD 223/2004 does not apply; and, if the law 14/2007 of biomedical research does apply, (i.e. if that is the applicable law), in part 2 of article 18 of this law, it stipulates that the performance of research that involves an invasive procedure in human beings will require the prior insurance for the damages and losses that might be derived from it for the person in whom they might be suffered. The present study does not involve any invasive procedure; therefore insurance is not required. | |

| 1.8 | PLAN FOR RELEASE AND DISSEMINATION |
| --- | --- |
| The quality of the plan for releasing and disseminating the results of the research project, by the form (through publications in scientific journals, patents, communications to conferences, etc.) and the place (at conferences, meetings, workshops for disclosure of results, etc.), will be assessed.  To our knowledge, there are no similar studies in our country. The research team has communicated to the managing board of the "Plan Integral de Oncología de Andalucia" a description of the research project put forward here; this Plan has agreed to feature as promoter and interested observer of the project.  We believe the results will justify their dissemination in scientific meetings of quality and in journals of national and international impact (at national and international conferences on screening by mammography, on Medical Oncology, on Healthcare Quality, and in national and international journals of Medical Oncology, of Cancer Prevention and of Healthcare Quality). | |

| **2. PRINCIPAL RESEARCHER AND RESEARCH TEAM** |
| --- |

| 2.1 | BRIEF SUMMARY OF THE RESEARCH GROUP FOR THE LAST 5 YEARS |
| --- | --- |
| | **Name and family names** | **Speciality** | **Type of researcher**  **(PI or CI)** | **Nº of years researching** | **Nº of articles in national journals** | **Nº of articles in international journals** | **Nº of Patents** | **Nº of contributions to national conferences** | **Nº of contributions to international conferences** | | --- | --- | --- | --- | --- | --- | --- | --- | --- | | José Manuel Baena Cañada | Medical Oncologist | Principal Researcher | 15 | 6 | 5 | 0 | 8 | 2 | | Encarnación Benítez Rodríguez | Preventive Medicine | Collaborating researcher | 12 | 7 | 3 | 0 | 6 | 0 | | Juan Nieto Vera | Preventive Medicine | Collaborating researcher | 15 | 3 | 1 |  |  |  | | Esperanza Arriola Arellano | Medical Oncologist | Collaborating researcher | 5 | 4 | 1 | 0 | 6 | 2 | | Patricia Ramírez Daffós | Medical Oncologist | Collaborating researcher | 3 | 2 | 0 | 0 | 11 | 9 | | Sara Estalella Mendoza | Medical Oncologist | Collaborating researcher | 1 | 4 | 0 | 0 | 4 | 0 | | Petra Rosado Varela | Medical Oncologist | Collaborating researcher | 1 | 3 | 0 | 0 | 1 | 0 | | |

| **3. MEANS AVAILABLE AND BUDGET REQUESTED** |
| --- |

| 3.1 | **MEANS AND RESOURCES AVAILABLE FOR CARRYING OUT THE PROJECT** |
| --- | --- |
| INVENTORIED MATERIAL The available resources of infrastructures are sufficient for the present clinical trial to be undertaken without any special difficulties with the means available in the Bay of Cadiz-La Janda Health District and in the Medical Oncology service of the Puerta del Mar University Hospital of Cadiz.   1. **BIBLIOGRAPHIC MATERIAL**   The Medical Oncology service has Internet access to be able to perform bibliographic searches and obtain the bibliographic material necessary. The "Puerta del Mar" Hospital also makes its library service available to researchers of the centre.   1. **PERSONNEL**   A sufficient number of women are attended in the centre of the Bay of Cadiz-La Janda Health District to be able to recruit the number of participants required to make up a sample of the correct size. The human resources and infrastructures available allow the present clinical trial to be undertaken without any special difficulties, provided the support personnel are contracted to perform the functions detailed in the “Plan of work”. As support to the research activity, the Medical Oncology service has available a Chemistry graduate to do the work of data manager, and a clinical trials nurse (both with contracts that will finish in December 2010), contracted by the Foundation for Research of the hospital. The researchers can also count on the Preventive Medicine service of the hospital for advice on methodology and statistical analysis, and the support and facilities of the Bay of Cadiz-La Janda Health District.In addition, the Plan Integral de Oncología de Andalucia has endorsed the present research project. | |

| 3.2 | **BUDGET REQUESTED AND JUSTIFICATION**.  Every section of the budget requested must be broken down and justified, indicating the items, units, unit price, etc., and, if the information is available, you are recommended to state the supplier**. In the event of not coinciding with the budget introduced in the computer application, the budget indicated there will take precedence** (See annex). | | | | |
| --- | --- | --- | --- | --- | --- |
| **ITEMS** | | **BUDGET REQUESTED** | | | |
|  |  | **YEAR 1** | **YEAR 2** | **YEAR 3** | **TOTAL** |
| **Goods and Services:**  **Inventoried equipment:**  Portable Computer  **Consumable Material:**  Stationery and office material, computer perishables (ink cartridges, etc.) | | 1,083.24  600  483.24 | 83.24  83.24 |  | 1,166.48  600  566.48 |
| **Personnel:**  **Support personnel for the research** | | 20,000  20,000 | 20,000  20,000 | 9,443.24  9,443.24 | 49,443.24  49,443.24 |
| **Other Expenditure:**  **Telephone quota** | | 360  360 | 360  360 |  | 720  720 |
| **TOTAL** | | **21,443.24** | **20,443.24** | **9,443.24** | **51,329.73** |
| Comments:  Inventoried: Portable Computer 600 €  Stationery and office material, computer perishables (ink cartridges, etc.) 483.24 € in the first year (*), and 83.24 € in the second  Telephone quota 30 € per month, 360 € per year, for the first two years  Support personnel for the research: 20,000 € in the first two years and 9,443.24 € in the third year (*) (differences based on the intensity of the activities programmed).  The financing requested is for the totality of the project. | | | | | |

| 3.3 | **DATA OF THE PERSONNEL REQUESTED** |
| --- | --- |
| Complete only in the event that personnel are requested.  **Type of personnel:** Support personnel  **Duration of the contract/grant:** 3 years  **Hours of dedication to the project:** 35 hours per week in the first 2 years, and 18 hours per week in the third year  **Activities to undertake in the project:** the support personnel will be responsible, together with the oncologists and residents of Oncology, for identifying the cases meeting the criteria for inclusion in the study, and this will be done actively by their physical presence in the centre of the mammography screening program, outside their normal hours of work. The support personnel (nurse and research assistant) will attend to all the visits made by the women to the centre of the screening program, and subsequently will be responsible for the telephone interviews one month later, and 2 years later. They will also be charged with the transcription of the data to a computer database, and with coordination with the epidemiologists.  **Justification of the need:** As support to the research activity, the Medical Oncology service has available a Chemistry graduate to do the work of data manager, and a clinical trials nurse, one contracted with a grant from the Instituto de Salud Carlos III – FIS grant - and the other with a grant from the Fundación para la Investigación Puerta del Mar de Cádiz, which will finish in December 2010. Without a new contract for support to the research, it will not be possible to undertake the project. | |

| **4. APPLICABILITY OF THE PROJECT FOR THE PUBLIC HEALTH SYSTEM OF ANDALUSIA** |
| --- |

| 4.1 | CLINICAL AND HEALTHCARE IMPACT /OR TECHNOLOGICAL DEVELOPMENT |
| --- | --- |
| Assessment will be made of the expectations for the transfer of research results to clinical practice, to technological innovation, to the organisation, to the management of resources and to the healthcare services or health policies, and in which the possible beneficiaries are described.  The real situation, which represents the point of departure, is the following: in all health systems, emphasis is given to the importance of the information that must be provided to the users for the clinical decision-making, giving priority to the principle of the user's autonomy; however, the secondary prevention of breast cancer by population mammography screening has developed in a context where the principle of authority prevails. That is, the woman is invited to participate because the health system proposes that it is for her benefit, but without giving her accurate data on those benefits and without presenting the risks. The object is to capture the highest possible proportion of those considered at risk. The ethics of this clinical approach in which the women are not informed is doubtful, to say the least. In this context, the introduction of improvements in the information given to the woman containing accurate data on the benefits and risks, will have a definite clinical impact and be of great healthcare value because it will be the woman herself who, after being capacitated to decide, will take her own decision based on both her own knowledge and her own values. It is to be seen if the improvement in the information is going to lead to a decrease in the rate of attendance (i.e. the capture), but, if it does, this should not be a cause for concern, since the woman will have decided freely. Thus the question to be answered by the present clinical assay (Does the level of knowledge of the women about the screening by mammography improve with an informed consent based on their appreciation of the benefits and risks?) is definitely relevant and will represent a benefit much appreciated by the women. It should therefore be considered a practical utility of prime importance in this area of healthcare. To our knowledge, there are no similar studies in our country. | |

| 4.2 | BIBLIOMETRIC IMPACT |
| --- | --- |
| State the relevance of the impact  We believe the results will justify their dissemination in scientific meetings of quality and in journals of national and international impact (at national and international conferences on screening by mammography, on Medical Oncology, on Healthcare Quality, and in national and international journals of Medical Oncology, of Cancer Prevention and of Healthcare Quality). We cannot accurately predict the specific journals and conferences that we will select for the publication and communication of the results, but their bibliometric impact will always be taken into account. | |

| 4.3 | GENERATION OF PATENTS |
| --- | --- |
| State the possible results that may be patentable.  Given that the model of informed consent is the property of the Nordic Cochrane Centre, which makes it available to researchers, it is not foreseen that this project will generate any possible patents. | |

**ANNEXES**

**Appendix 2. Annexes**

**Annex 1. Official information by letter**

### Primary Attention District Bahía de Cádiz-La Janda

Unit of Exploration

Avda. Ramón de Carranza Nº 19

11006 – Cádiz Telephone: 956-00.47.50

Dear Madam:

As you will already know, the Program for Early Detection of Breast Cancer is being carried out in this District Health. A mammography, which is the basic test applied in this Program of Prevention, should be done every two years, for all women from 45 to 69 years of age. We are writing to tell you that you have an appointment for the performance of a mammography, in the Early Detection Unit of this Healthcare District Bahía de Cádiz - La Janda, located in the Avda. Ramón de Carranza, Nº19.

We would like to remind you of the importance of this test for the early diagnosis of possible anomalies in the breast, since gaining time for the appropriate treatment can be decisive.

To make everything easier for you, we would ask you to follow the recommendations listed here:

| - *Bring your Social Security Healthcare Card, or a copy of the written request for it, if you have not yet received it.* - *Bring also your* ***DNI*** *(identity card), because although you may not belong to the Social Security, with this document you will be attended.* - *If the time and day of the appointment are not convenient, telephone to obtain another appointment, to the number indicated above (from 9.00 to 20.00).* - *If you have had a mammography done not long ago, please attend the appointment and bring the result with you. If you do not have the result, please still attend the appointment.* - *Wear comfortable clothing, easy to take off and put back on.* - *Do not put any substance on your breasts, such as creams, talcum powder, oils, etc.* |
| --- |

Even if you have previously had mammographies done, it is important to continue attending the appointments until you reach 69 years of age.

Please understand when we insist that it is in your own interest, for your future health, that you should attend this appointment for screening that we offer you.

With our very best wishes,

THE DIRECTOR OF THE

BAHIA DE CADIZ - LA JANDA DISTRICT

**Annex 2. Experimental information**

This information was based on the 1st edition (2008) of the document created by the Nordic Cochrane Centre, Copenhagen (Denmark), a Spanish translation of which can be consulted in the following Websites: www.screening.dk and www.cochrane.dk

**Annex 3. Sources that you can access to request information:**

- Your primary care doctor.
- Servicio Andaluz de Salud: <http://www.juntadeandalucia.es/servicioandaluzdesalud/principal/documentosAcc.asp?pagina=gr_serviciossanitarios1_2_10>
- Early Detection of Breast Cancer in Andalusia. Dirección General de Asistencia Sanitaria. Servicio Andaluz de Salud. Consejería de Salud Avda. de la Constitución nº 18 41071 SEVILLA EMAIL: [rosario.fernandez.echegaray.sspa@juntadeandalucia.es](mailto:rosario.fernandez.echegaray.sspa@juntadeandalucia.es)
- Portal endorsed by the Plan Integral de Oncología de Andalucía (PIOA): <http://www.onconocimiento.net/>
- Website of Programas de Cáncer de Mama (PCM): <http://ppc.cesga.es/>
- Asociación Española contra el Cáncer. C/ Brunete, 4 11007 Cádiz
  956 281 164: [www.aecc.es](http://www.aecc.es/)
- Asociación Gaditana de Mujeres con Cáncer de Mama (AGAMAMA). C/ Sociedad, 3 11008 Cádiz.
  How to get there:

956 266 266Service Area:  -

Category:

Company not verified

- : <http://www.agamama.org/que-es-agamama>
- Federación Española de Cáncer de Mama: <http://fecma.vinagrero.es/Default.aspx>
- Sociedad Española de Oncología Médica (SEOM): [www.seom.org](http://www.seom.org/)

**ANNEX 4. QUESTIONS ON THE PROGRAM OF SCREENING BY MAMMOGRAPHY AND METHOD OF ASSESSMENT** (i.e. Questions about level of knowledge of participants)

1. A mammography performed in a screening program. What does it mean?
   - A mammography done for women when they are healthy.
   - A mammography done for women when they notice a lump or other changes in the breasts.
   - I am not sure.
2. Imagine 2000 women who have mammographies done regularly for 10 years. How many deaths from breast cancer will be saved thanks to the early detection?

....................... out of those 2000

1. Can screening by mammography detect all kinds of breast cancer?
   - Yes
   - No
   - I am not sure.
2. Are all the cancers diagnosed in a screening by mammography really malignant tumours that would cause the death of the woman if not diagnosed and treated?
   - Yes
   - No
   - I am not sure.
3. If 2000 women have mammographies done regularly for 10 years, how many of them will be diagnosed and treated unnecessarily because a pseudo-cancerous lesion is detected that would never become a tumour?

....................... out of those 2000

1. Will all the women for whom the screening by mammography shows some alteration in the breast be diagnosed with cancer?
   - Yes
   - No
   - I am not sure.
2. If 2000 women have mammographies done regularly for 10 years, how many of them will be affected by a false alarm that would oblige them to have other tests done before they are finally diagnosed as not having cancer?

....................... out of those 2000.

**Guide for marking:**

If the answer is correct, score with 1 point the questions with three possible options, and with 2 points the quantitative questions with only one response option.

**Annex 5. QUESTIONNAIRE ON THE SOURCES OF INFORMATION**

**Please indicate how frequently you have received information about the mammography screening program from the following sources:**

**Family and friends:**

- Never
- Rarely
- Sometimes
- Frequently
- Don't know

**Experts:**

**Family doctor:**

- Never
- Rarely
- Sometimes
- Frequently
- Don't know

**Pharmacist:**

- Never
- Rarely
- Sometimes
- Frequently
- Don't know

**Communications media:**

**Television:**

- Never
- Rarely
- Sometimes
- Frequently
- Don't know

**Radio:**

- Never
- Rarely
- Sometimes
- Frequently
- Don't know

**Newspapers:**

- Never
- Rarely
- Sometimes
- Frequently
- Don't know

**Healthcare services and institutions:**

**Documentation from the SAS (Health Service of Andalusia):**

- Never
- Rarely
- Sometimes
- Frequently
- Don't know

**Documentation from the Ministry of Health:**

- Never
- Rarely
- Sometimes
- Frequently
- Don't know

**Documentation from insurance companies:**

- Never
- Rarely
- Sometimes
- Frequently
- Don't know

**Documentation from Consumers Associations:**

- Never
- Rarely
- Sometimes
- Frequently
- Don't know

**Documentation from self-help associations (e.g. Agamama, AECC):**

- Never
- Rarely
- Sometimes
- Frequently
- Don't know

**Books:**

- Never
- Rarely
- Sometimes
- Frequently
- Don't know

**Internet (Web pages of health and medicine):**

- Never
- Rarely
- Sometimes
- Frequently
- Don't know

# Annex 6. Assessment of the attitude towards the program of screening by mammography

In the following questions, please put a circle around the number from 0 to 6 that best applies to yourself:

1. **For you the screening by mammography is:**

0 1 2 3 4 5 6

A good option A bad option

1. **For you the screening by mammography is:**

0 1 2 3 4 5 6

Beneficial Harmful

1. **For you the screening by mammography is:**

0 1 2 3 4 5 6

Important Not at all important

1. **For you the screening by mammography is:**

0 1 2 3 4 5 6

Pleasant Unpleasant

**Annex 7. QUESTIONNAIRE ON THE DECISION TO PARTICIPATE IN THE MAMMOGRAPHY SCREENING**

**With the information that has been given to you, what is your decision on participating in the program of screening by mammography?**

- I have decided to participate
- I have decided not to participate
- I am undecided

**Annex 8. QUESTIONNAIRE FOR ASSESSMENT OF THE ANXIETY AND DEPRESSION RELATED TO THE INFORMATION ON MAMMOGRAPHY PROVIDED**

# HOSPITAL ANXIETY AND DEPRESSION SCALE

*This questionnaire has been designed to help us know how you feel in relation to the performance of the screening mammography. Read each sentence and mark the answer that best matches how you have felt during the past week. Do not think too much about your answers. It is more likely that if you answer quickly, your answers will reflect more closely how you have really been feeling.*

**1. I feel tense or nervous.**

􀂉 All day long

􀂉 Almost all the day

􀂉 Sometimes

􀂉 Never

**2. I continue enjoying the same things as always.**

􀂉 Never

􀂉 Rarely

􀂉 Frequently

􀂉 Almost always

**3. I feel a kind of fear, as if something were going to happen to me.**

􀂉 Yes, and very intensely.

􀂉 Yes, but not very intensely.

􀂉 Yes, but it does not bother me.

􀂉 No, I don't feel anything like that.

**4. I am able to laugh and see the amusing side of things.**

􀂉 Never

􀂉 Rarely

􀂉 Frequently

􀂉 Almost always

**5. I have my head full of worries.**

􀂉 All day long

􀂉 Almost all the day

􀂉 Sometimes

􀂉 Never

**6. I feel happy.**

􀂉 Never

􀂉 Hardly ever

􀂉 Sometimes

􀂉 Almost always

**7. I can be sat down comfortably and feel relaxed.**

􀂉 Never

􀂉 Hardly ever

􀂉 Sometimes

􀂉 Almost always

**8. I feel I am sluggish and slow to react.**

􀂉 All day long

􀂉 Almost all the day

􀂉 Sometimes

􀂉 Never

**9. I experience an unpleasant sensation of nerves and emptiness inside.**

􀂉 Almost always

􀂉 Frequently

􀂉 Rarely

􀂉 Never

**10. I have lost interest in my personal appearance.**

􀂉 Totally

􀂉 Rather

􀂉 A little

􀂉 Not at all

**11. I feel unsettled, as if I could not keep still.**

􀂉 Very much

􀂉 Rather

􀂉 A little

􀂉 Not at all

**12. I look forward to things eagerly.**

􀂉 Never

􀂉 Rarely

􀂉 Frequently

􀂉 Almost always

**13. I get sudden attacks of panicky feelings.**

􀂉 Very frequently

􀂉 Sometimes

􀂉 Hardly ever

􀂉 Never

**14. I get enjoyment from a good book, the radio, or a television program.**

􀂉 Never

􀂉 Rarely

􀂉 Almost always

􀂉 Always

**Annex 9. QUESTIONNAIRE FOR ASSESSMENT OF WORRIES ABOUT CANCER**

1. During the past month, how frequently have you thought about your chances of developing cancer? Would you say.... (please tick only one box for your answer):

Never or on very few occasions

Some times

Often

Almost all the time

1. During the past month, when thinking about the possibility of developing cancer, has this affected the way you feel? Would you say....

Never or on very few occasions

Some times

Often

Almost all the time

1. During the past month, when thinking about the possibility of developing cancer, has this affected your capacity to carry out your day-to-day activities? Would you say...

Never or on very few occasions

Some times

Often

Almost all the time

1. How worried are you that one day you might develop cancer? Would you say....

Not at all

A little

Fairly

Very much.

1. How frequently do you worry about the possibility of developing cancer? Would you say ......

Never or on very few occasions

Some times

Frequently

Constantly.

1. Is being worried about developing cancer a serious problem for you? Would you say...

No, not at all

A little

It is clearly a problem

Yes, it is a very serious problem
